# Dr. Bedford Brown

**Published:** 1897-11

**Authors:** 


DR. BEDFORD BROWN, of Alexandria, Va., died at his home in that city September 12, 1897, aged 76 years. He was born in North

Carolina and was the son of Senator Bedford Brown, who represented North Carolina in the senate many years ago. He began the practice of his profession in his native state, and at the outset of the civil war, in 1861, he entered the confederate army as a medical officer, where he rose to the rank of medical director. After the war he settled at Alexandria, Va., where he continued to reside until his death. He was a member of the Medical Society of the State of Virginia, president in 1886; of the Southern Surgical and Gynecological Association, president in 1893; member of the first Pan-American Medical Congress; of the American Medical Association, and honorary member of the Medical Society of the District of Columbia. He had also been a member of the Virginia State Medical Examining Board.

Dr. Brown was regarded with high esteem throughout the south, and very justly so, for he was a man of erudition, amiability of disposition, ability in his profession and spotless in his character. He will be greatly missed among his many friends and acquaintances, and especially in the Southern Surgical and Gynecological Association, where he was a conspicuous figure for many years.

